# Irreverent Nature of Dissymmetry Factor and Quantum Yield in Circularly Polarized Luminescence of Small Organic Molecules

**DOI:** 10.3389/fchem.2020.00448

**Published:** 2020-06-09

**Authors:** Yuya Nagata, Tadashi Mori

**Affiliations:** ^1^Institute for Chemical Reaction Design and Discovery (WPI-ICReDD), Hokkaido University, Sapporo, Japan; ^2^Department of Applied Chemistry, Graduate School of Engineering, Osaka University, Osaka, Japan

**Keywords:** dissymmetry factor, luminescence quantum yield, circularly polarized luminescence, structure-chiroptical property relationship, allowed π-π^*^ transition

## Abstract

Recently, a rational modification of small organic molecules has attracted considerable attention for designing advanced materials with enhanced circularly polarized luminescence (CPL) activity. A particular emphasis has been placed on fully allowed π-π^*^ transition of rigid aromatic systems, due to their relatively superior emission properties or quantum yields of luminescence (Φ_lum_). However, their dissymmetry factors (*g*_lum_), differential left and right CPL intensities, are typically disappointingly low at least in one to two orders of magnitude. Truly useful organic CPL materials, rated by a circular polarization luminosity index (Λ_CPL_) per single molecule, possess both |*g*_lum_| and Φ_lum_ values high. However, how to improve these two factors simultaneously with a proper molecular design is an open question. Here, we addressed this issue by theoretical and statistical inspection on a possible relation of the *g*_lum_ and Φ_lum_ values. According to the analysis, we propose simple, unpretentious, yet pertinent guidelines for designing superior organic CPL materials for the future with large Λ_CPL_ values.

## Introduction

An increasingly considerable attention has been paid recently to circularly polarized luminescence (CPL) behavior (Riehl and Muller, 2011; Longhi et al., 2016). Not only their potential applications in chemical sensors (Bradberry et al., 2014), biological probes (Muller, 2009), and three-dimensional displays (Schadt, 1997), but also the exclusive chiroptical and photophysical property of CPL reflects the structural information of chiral molecules or molecules in chiral environment in their excited states (Richardson and Riehl, 1977; Riehl and Richardson, 1986). Every so often, the CPL signal is relatively weak but can be unique and selective; accordingly, the CPL materials are believed to be applicable to various smarter photonic materials and discerning biological censors (Han et al., 2018; Ma et al., 2019). At an early stage, the materials had been developed for derivatives of lanthanoids, due to their intrinsic characteristics that electronic forbidden f–f transitions commonly afford better dissymmetry factor (or a degree of chirality, *g*_lum_, vide infra) (Carr et al., 2012; Zinna and Di Bari, 2015). Recent advance in supramolecular chirality is another trend in the CPL chemistry, where improved responses have been frequently reported through molecular aggregation, agglomeration, flocculation, as well as their combinations (Kumar et al., 2015; Sang et al., 2020). However, systematic investigations to pursue a so-called structure–property relationship to attain a reliable strategy and a design principle for the superior CPL materials, even for more simple isolated molecular systems, have been quite limited. As such, current studies on the CPL materials are mostly based on a cut-and-try basis. Further discussions and many examples are available in recent review articles (Sanchez-Carnerero et al., 2015; Tanaka et al., 2018b).

Naturally, an observed difference between emission intensities of left- and right-handed circularly polarized light (*I*_L_-*I*_R_) at a given frequency ν in the CPL measurement on chiral substance depends on an intensity of an incident excitation light. Therefore, a degree of chirality in the CPL response is generally discussed with the polarization efficiency, or the dissymmetry factor of luminescence (*g*_lum_). Thus, the *g*_lum_ value is a difference emission intensity divided by an averaged intensity at a given frequency ν, which is defined as follows:

(1) $$\begin{array}{l} g_{\text{lum}}\left(\nu \right)=2\;\frac{I_{L}\left(\nu \right)-I_{R}\left(\nu \right)}{I_{L}\left(\nu \right)+I_{R}\left(\nu \right)} \end{array}$$

By definition, minimum and maximum *g*_lum_ factors are −2 and +2. Most of the studies that explore better CPL molecules thus pursue molecules with larger absolute *g*_lum_ value (i.e., |*g*_lum_|), as this parameter is frequently the most limiting factor, particularly in small organic molecules where *g*_lum_ factors are typically as low as in an order of 10^−5^ to 10^−3^ range.

In the 1960s and 1970s, the CPL chemistry on small organic molecules had been limited to constraint cyclic ketones, where relatively larger *g*_lum_ values were obtained due to the electronically forbidden n–π^*^ transition. In most of these molecules, chiral distortion of carbonyl moiety is usually released in their excited states, the degree of which is highly dependent on the nature of the molecule. Accordingly, *g*_lum_ prediction of chiral ketones is specifically challenging. Recent emphasis has been rather placed on electronically allowed π-π^*^ transition of rigid aromatic systems, for reasons such as below. Firstly, these systems often afford much improved fluorescence probability. Second, a facile structural modification is conceivable that can fine-tune absorption and emission wavelengths and bandwidths, and possibly the degree of dissymmetry as well. Also, the degree of excited-state relaxation in such systems has been found surprisingly systematic, although slightly dependent on the structural motifs or types of chirality. Such statistical analyses afforded empirical linear correlations between the dissymmetry factors of luminescence and absorption, allowing us an empirical yet a rational design (Tanaka et al., 2018b).

**Figure 1:**
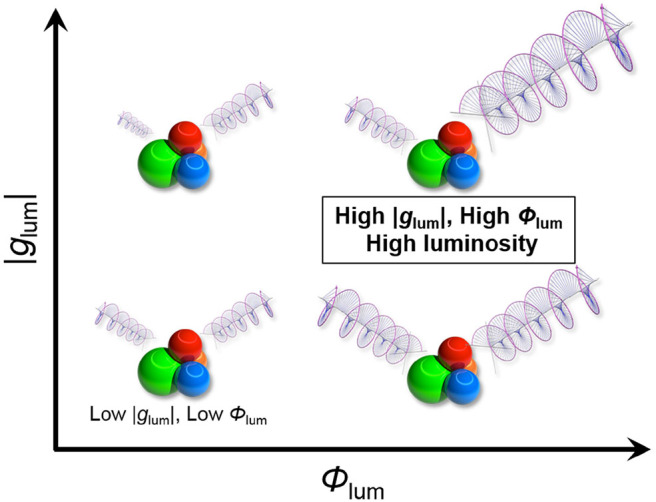
Conceptual diagram for two-dimensional improvement of CPL materials.

In order to fully understand the overall CPL efficiency, other photophysical parameters beside the dissymmetry factor (*g*_lum_) should be also taken into consideration (Figure 1). As more materials-oriented intrinsic index of CPL efficiency, we propose a circular polarization luminosity (Λ_CPL_) per single chiral molecule in the excited state, which is defined as follows:

(2) $$\begin{array}{l} \Lambda_{\text{CPL}}=f\times \Phi_{\text{lum}}\times \frac{\left|g_{lum}\right|}{2} \end{array}$$

where *f* and Φ_lum_ are efficiencies of light absorption (oscillator strength) and emission intensity (quantum yield), respectively. By definition, minimum and maximum Λ_CPL_ values are 0 and 1. Hypothetically, molecules with larger Λ_CPL_ values at desired excitation and emission wavelengths are considered as satisfactory chiroptical materials. In the following discussion, we made an effort to understand a possible relationship among the photophysical parameters in the CPL behavior, particularly that between *g*_lum_ and Φ_lum_.

## Theoretical Consideration

In real spectra, the CPL and fluorescence bands are characterized by distinct parameters, which are called rotational (*R*) and dipole (*D*) strengths, respectively. In isotropic solution, the following equations generally fold (in cgs unit) for the CPL (*I*_L_-*I*_R_) and total (*I*_L_ + *I*_R_) emission intensities from chiral substance as a function of ν:

(3) $$\begin{array}{l} I_{L}\left(\nu \right)-I_{R}\left(\nu \right)=\frac{16{\;\nu }^{3}\;\rho \left(\nu \right)}{3\;c^{3}\;\hbar^{4}}\;R \end{array}$$

(4) $$\begin{array}{l} I_{L}\left(\nu \right)+I_{R}\left(\nu \right)=\frac{8{\;\nu }^{3}\;\rho \left(\nu \right)}{3\;c^{3}\;\hbar^{4}}\;D \end{array}$$

where *h* is the reduced Planck's constant, *c* is the speed of light, and ρ(ν) is a Gaussian band shape.

Theoretically, the value *D* is defined as the square of an electric transition dipole moment (**μ**) for an electronic transition between an emissive state *j* and a ground state *i*:

(5) $$\begin{array}{l} D={\langle {\text{Ψ}}_{j}|\mu |{\text{Ψ}}_{i}\rangle }^{2} \end{array}$$

According to Rosenfeld (Rosenfeld, 1929), the value *R* can be expressed as a product of wavefunction overlap integrals between the electric (**μ**) and magnetic (***m***) transition dipole moments, as follows:

(6) $$\begin{array}{l} R=\text{lm}\left[\langle {\text{Ψ}}_{j}|\mu |{\text{Ψ}}_{i}\rangle \cdot \langle {\text{Ψ}}_{i}|m|{\text{Ψ}}_{j}\rangle \right] \end{array}$$

where Im refers to an imaginary component of the scalar product between real vector **μ** and imaginary vector ***m***. In most situations, this is also expressed as:

(7) $$\begin{array}{l} R=\;|\mu ||m|\cos\theta \end{array}$$

where θ is the angle between the two dipole moments. This obviously demonstrates a non-orthogonal nature of **μ** and ***m*** of chiral materials. By substituting Equations (3) and (4) for Equation (1), the dissymmetry factor can be simplified with *R* and *D* as follows:

(8) $$\begin{array}{l} g_{lum}=4\times \frac{R}{D} \end{array}$$

Equation (8) clearly suggests the linear correlation between the *g*_lum_ value against the inverse of *D*. That is, *g*_lum_ should be reciprocally proportional to the square of transition probability, if the value *R* is independent to *D*. This is empirically in accord with the fact that classical examples of CPL responsive materials were based on the molecules with forbidden transitions, in which better *g*_lum_ factors were frequently reported. In a different expression, Equation (8) is also stated as:

(9) $$\begin{array}{l} g_{lum}=4\times \frac{|m|\cos\theta }{\left|\mu \right|} \end{array}$$

Thus, *g*_lum_ is proportional to reciprocal amplitude of **μ**, under conditions that **μ** is independent to ***m***. For further details on the relevant theoretical consideration and numerical expressions, see refs (Richardson and Riehl, 1977) and (Riehl and Richardson, 1986).

The quantum yield of emission Φ_lum_ is determined by the rate of radiative and non-radiative decays, as follows:

(10) $$\begin{array}{l} \Phi_{\text{lum}}=\frac{k_{r}}{k_{r}+k_{nr}}=\frac{1}{1-\left(-\frac{k_{nr}}{k_{r}}\right)} \end{array}$$

In order to assess a possible correlation between *g*_lum_ and Φ_lum_ values, we may consider the following relations (Carr et al., 2012; Kumar et al., 2015; Tanaka et al., 2018b; Sang et al., 2020) that are valid only at the condition of *k*_nr_/*k*_f_ ≪ 1, for which the molecules have relatively good emission properties. This allows to expand the Equation (10) to:

(11) $$\begin{array}{l} \Phi_{\text{lum}}\approx 1-\frac{k_{nr}}{k_{r}},\text{ in the limit of }\frac{k_{nr}}{k_{r}}\ll 1 \end{array}$$

The rate of emission is dependent on *f* and the square of the frequency of the electronic transition ν. Here, we ignore the difference between absorption and emission processes as structural relaxation in the excited state can be negligible in rigid aromatic systems. Also, the experimental emission and absorption intensities are proportional to the square of corresponding electric transition dipole moments. Thus, Φ_lum_ is related to the electronic transition dipole moment **μ** as follows:

(12) $$\begin{array}{l} {1-\Phi }_{lum}\propto |\mu {|}^{-2} \end{array}$$

Subsequently, Equations (9) and (12) can be rearranged to:

(13) $$\begin{array}{l} g_{lum}\propto |m|\cos\theta \times \sqrt{{1-\Phi }_{\text{lum}}} \end{array}$$

Therefore, under the condition that Φ_lum_ and ***m*** can be regarded independent, *g*_lum_ values are dependent to the square of (1 – Φ_lum_). That is:

(14) $$\begin{array}{l} g_{lum}\propto \sqrt{{1-\Phi }_{\text{lum}}},\text{ in the limit of }\frac{k_{nr}}{k_{r}}\ll 1 \end{array}$$

Finally, Equation (2) can be also reorganized into:

(15) $$\begin{array}{l} \Lambda_{\text{CPL}}\propto |\mu {|}^{2}\times \Phi_{\text{lum}}\times \frac{|m|\cos\theta }{\left|\mu \right|}=|\mu ||m|\cos\theta \times \Phi_{\text{lum}}\\ =R\times \Phi_{\text{lum}} \end{array}$$

Thus, as the first approximation, the circular polarization luminosity (Λ_CPL_), a key parameter for the excellent CPL materials, is eventually related to the rotational strength (*R*) and the emission quantum yield (Φ_lum_). Note that this equation holds without the condition of *k*_nr_/*k*_f_ ≪ 1.

## Statistical Analyses

**Figure 2:**
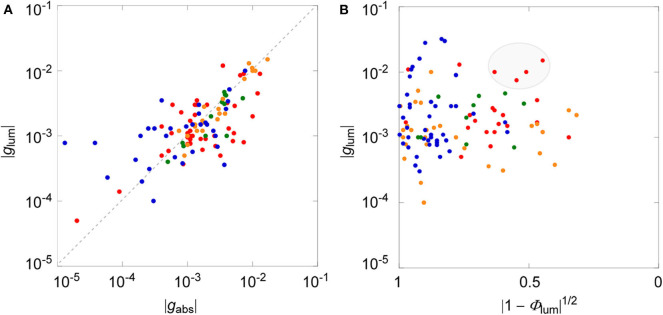
**(A)** Log–log plot between luminescence (*g*_lum_) and absorption (*g*_abs_) dissymmetry factors. **(B)** Log plot between *g*_lum_ and square of 1—luminescence quantum yield (Φ_lum_). Blue, helicenes and derivatives; red, planar chiral cyclophanes; orange, binaphthyls with axial chirality; green chiral BODIPY derivatives. Data are taken from Tanaka et al. (2018b).

We have recently collected all the reported CPL data that were associated with circular dichroisms (CDs) for small organic molecules up to the year 2017 (Tanaka et al., 2018b). We found the direct correlation between dissymmetry factors of luminescence and absorption, affording an empirical linear correlation of |*g*_lum_| = 0.81 × |*g*_abs_| (*r*^2^ = 0.60) as a global fit for all the CPL and CD data of the electronically allowed π-π^*^ transition of rigid aromatic systems. For comparison and clarity, the same data were plotted in log–log format depicted in Figure 2A. Although some scattered data were apparent, statistical analysis led to the same conclusion that there is a linear correlation between two dissymmetry factors with a slop of ≈1. As discussed above, the *g*_lum_ value will be correlated to the square of (1 – Φ_lum_), unless there is extensive bias. Such an analysis was performed as shown in Figure 2B, utilizing all the chiral molecules used in the same review (Tanaka et al., 2018b). As clearly seen, data were more dispersed and direct correlation was not obtained between *g*_lum_ and Φ_lum_, at least among these samples, both in global plot and among sub-class of chirality, which was categorized in different colors.

## Discussion

Before providing our supposition on the above observations, we better comment on a limitation of our evaluations. Possible issues on reliability of our data analyses may include the following: (1) Limiting examples: Our analyses were rather limited in terms of numbers of available data (*N* ≈ 100) in indefectible statistical point of view. (2) Exclusion of negative data: In particular, data with low Φ_lum_ values (<10^−3^) are almost completely neglected as such systems have been rarely published. Such trend is also true for those with low *g*_lum_ values (<10^−5^). (3) Measurement conditions: Measurements to be compared are better to be identical. Also, both the CPL and luminescence experiments should be executed under the comparable conditions. From time to time, Φ_lum_ and *g*_lum_ were evaluated at specific peak wavelengths that were not always matching each other (e.g., *g*_lum_ was reported at a relatively feeble shoulder position of emission). In such cases, both parameters are maximized at individual wavelengths, but the correlation may be lost, or at least deteriorated. In other instances, very wide slit widths are employed in the CPL experiment. The *g*_lum_ values tend to be observed smaller as widths of slit (window of light propagation) in the CPL spectroscopy are increased. This is often inevitable, however, for samples of weak signal (i.e., low luminescence and/or dissymmetry factor). (4) Sample quality: As well as the purity and optical purity of the chiral samples, additional experimental issues such as aggregation, band overlap, and vibronic contribution should be carefully considered and possibly be eliminated or corrected. Such propositions, however, have been overlooked in most of the reports.

Although we admit that more studies are certainly needed to have a definite relevancy between *g*_lum_ and Φ_lum_ values, we may deduce the following (tentative) suppositions.

1. The plots between *g*_lum_ and Φ_lum_ values, even among the sub-class of different types of chiral molecules, provided substantially dispersed ones (Figure 2B). They did not provide a linear (or any meaningful) relationship, as has been expected by theory or by intuition from the classical examples. This immediately indicates that there is some bias between these values. We believe that this is due, at least in part, to the fact that the data of low Φ_lum_ values were missing simply because such numbers were reluctant to be included in a publication. In fact, the plots based on the reported values were widely dispersed with the exception of a region with low Φ_lum_ values (right-hand side).
2. At a first glance, the fact that there was no immediate correlation between *g*_lum_ and Φ_lum_ values was somewhat disappointing. However, it was also realized that the *g*_lum_ values were still expanded in a whole range between 10^−5^ and 10^−1^ within the selected Φ_lum_ domain. This observation clearly infers that the *g*_lum_ values may be improved irrespective to the emission property, in spite of possible correlation in Equation (14). As such, we suggest the rather straightforward strategy for designing superior CPL materials having better circular polarization luminosity (Λ_CPL_), that is, to inspect a systematic structural modification on certain molecules already demonstrating high Φ_lum_ value in a trial-and-error manner.
3. In this regard, we can point to some data located at the top-middle position in Figure 2B (highlighted in a gray ellipse), those simultaneously possessing relatively larger *g*_lum_ and Φ_lum_ values. These include some planar chiral rigid cyclophane derivatives that may be quite promising as the starting points for more improved CPL materials with larger *g*_lum_ value concomitantly keeping high Φ_lum_ value. In another respect, it is worth noting that there have been substantially growing numbers of investigations recently that report the improved CPL responses based on molecular symmetry with helicene derivatives (Tanaka et al., 2018a,c; Isla et al., 2019; Schaack et al., 2019; Zhao et al., 2019).

## Concluding Remarks

Although the number of reported examples of CPL active small organic molecules has been rapidly increasing, mostly being explored in a cut-and-try basis, a structure–property relationship that can guide the design principle has not been established. Thus, to design desired CPL response in molecular systems is still challenging. In most of the CPL studies in organic molecules, the dissymmetry factor of luminescence (*g*_lum_) is the limiting factor because the reported values are considerably smaller, usually in several orders of magnitude than the limiting value. Alternatively, other photophysical parameters, in particular the luminescence quantum yields (Φ_lum_), are also an important factor to develop the truly useful CPL materials.

In this contribution, we tried to determine the possible correlation between *g*_lum_ and Φ_lum_ values, both in theoretical formula and experimental observations. It was a little surprise that the observed *g*_lum_ values of π-π^*^ transition of rigid aromatic molecules are independent to their Φ_lum_ values, despite the expected correlation derived from Equation (14). Rather, a direct relationship between the circular polarization luminosity (Λ_CPL_) and rotational strength (*R*) found in Equation (15) seems more substantial. Our analyses also advocate that the *g*_lum_ values can be improved irrespective to Φ_lum_. Therefore, the most straightforward means to develop the improved CPL materials could be a methodical structural modification of aromatic systems that already enjoy the high Φ_lum_ values, which will directly maximize the circular polarization luminosity (Λ_CPL_), the real measure for the superior CPL materials. We hope our analyses and suppositions may be of benefit for future design of materials of better CPL responses.

## Author Contributions

TM wrote the first draft of the manuscript. Both authors contributed to manuscript revision, and read and approved the submitted version.

## Conflict of Interest

The authors declare that the research was conducted in the absence of any commercial or financial relationships that could be construed as a potential conflict of interest. The reviewer CY declared a past co-authorship with one of the author TM to the handling editor.
